# Omnidirectional Sensory and Motor Volumes in Electric Fish

**DOI:** 10.1371/journal.pbio.0050301

**Published:** 2007-11-13

**Authors:** James B Snyder, Mark E Nelson, Joel W Burdick, Malcolm A MacIver

**Affiliations:** 1 Department of Biomedical Engineering, R.R. McCormick School of Engineering and Applied Science, Northwestern University, Evanston, Illinois, United States of America; 2 Department of Molecular and Integrative Physiology and the Beckman Institute for Advanced Science and Technology, University of Illinois, Urbana, Illinois, United States of America; 3 Division of Engineering and Applied Science, California Institute of Technology, Pasadena, California, United States of America; 4 Department of Mechanical Engineering, R.R. McCormick School of Engineering and Applied Science, and Department of Neurobiology and Physiology, Northwestern University, Evanston, Illinois, United States of America; University of Ottawa, Canada

## Abstract

Active sensing organisms, such as bats, dolphins, and weakly electric fish, generate a 3-D space for active sensation by emitting self-generated energy into the environment. For a weakly electric fish, we demonstrate that the electrosensory space for prey detection has an unusual, omnidirectional shape. We compare this sensory volume with the animal's motor volume—the volume swept out by the body over selected time intervals and over the time it takes to come to a stop from typical hunting velocities. We find that the motor volume has a similar omnidirectional shape, which can be attributed to the fish's backward-swimming capabilities and body dynamics. We assessed the electrosensory space for prey detection by analyzing simulated changes in spiking activity of primary electrosensory afferents during empirically measured and synthetic prey capture trials. The animal's motor volume was reconstructed from video recordings of body motion during prey capture behavior. Our results suggest that in weakly electric fish, there is a close connection between the shape of the sensory and motor volumes. We consider three general spatial relationships between 3-D sensory and motor volumes in active and passive-sensing animals, and we examine hypotheses about these relationships in the context of the volumes we quantify for weakly electric fish. We propose that the ratio of the sensory volume to the motor volume provides insight into behavioral control strategies across all animals.

## Author Summary

Most animals, including humans, have sensory and motor capabilities that are biased in the forward direction. The black ghost knifefish, a nocturnal, weakly electric fish from the Amazon, is an interesting exception to this general rule. We demonstrate that these fish have sensing and motor capabilities that are omnidirectional. By combining video analysis of prey capture trajectories with computational modeling of the fish's electrosensory capabilities, we were able to quantify and compare the 3-D volumes for sensation and movement. We found that the volume in which prey are detected is similar in size to the volume needed by the fish to stop. We suggest that this coupling may arise from constraints that the animal faces when using self-generated energy to probe its environment. This is similar to the way in which the angular coverage and range of an automobile's headlights are designed to match certain motion characteristics of the vehicle, such as its typical cruising speed, turning angle, and stopping distance. We suggest that the degree of overlap between sensory and movement volumes can provide insight into the types of control strategies that are best suited for guiding behavior.

## Abstract 3

Most animals have sensory and motor capabilities that are biased in the forward direction. The black ghost, a nocturnal weakly electric fish, is an exception to this general rule. We demonstrate that these fish have omnidirectional sensing and motor capabilities.

## Introduction

Bats, dolphins, and electric fish are well-known examples of animals that emit energy into the environment for the purpose of sensing their surroundings. We refer to these as “active” sensing systems [1], to distinguish them from “passive” systems that rely on extrinsic sources of energy, such as sunlight. For an active-sensing organism, the intensity and spatial profile of the emitted probe energy influences the volume of space within which target objects can be detected, which we refer to as the sensory volume. In the context of a foraging task with sparsely distributed targets, the probability of target detection and thus overall foraging efficiency generally increases as the size of the sensory volume increases. There are also a number of factors that may potentially limit the extent of the sensory volume of active sensory systems beyond the metabolic costs associated with the encoding and neural processing of sensory information, common to both active and passive systems [2]. One constraint is related to the metabolic cost of energy emission, which scales steeply with sensing range. In general, both the outbound probe energy and the return signal are subject to the inverse-square dependence of spherical spreading loss, which means that the strength of the return signal falls as 1/*r* ^4^, where *r* is the distance to the target. For the general case, doubling the active sensing range would require a 16-fold (2^4^) increase in emitted energy [1,3].

Another constraint is imposed by the interaction of the emitted probe energy with nearby nontarget objects, which we refer to generically as clutter. For example, when a bat forages for flying insects near vegetation, the reflected signal from leaves and branches can be much more intense than the signal from the prey [4]. Backscattered energy from nontarget objects can impose a substantial constraint on the maximum probe intensity that can be used without saturating the sensory receptors. The degree of clutter in the habitat will likely influence the desirable emission power and beam spread, and thus affect the size and shape of the active sensory volume. Dolphins are known to reduce their emission power by 40 dB in concrete tanks [5] versus open pens [6]. Bats that glean their prey from surfaces in cluttered environments produce weak echolocation calls and are sometimes referred to as whispering bats [4,7]. Bats that forage in open areas produce calls that are 40–50 dB more intense, and they decrease call intensity during the terminal phase as they near their target [8,9].

**Figure 1:**
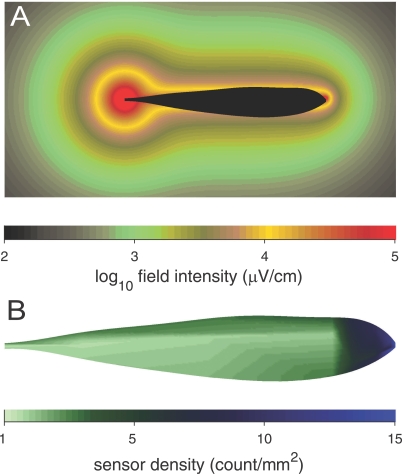
Spatial Distributions in the Active Electrosensory System of A. albifrons (A) Magnitude of the self-generated electric field in water of 35-μS/cm conductivity from an empirically validated computational model [41]. (B) Density of tuberous (active) electroreceptor sense organs (interpolated from measurements in [44]). The fish is shown without fins because there are no electroreceptors on the fins.

**Figure 2:**
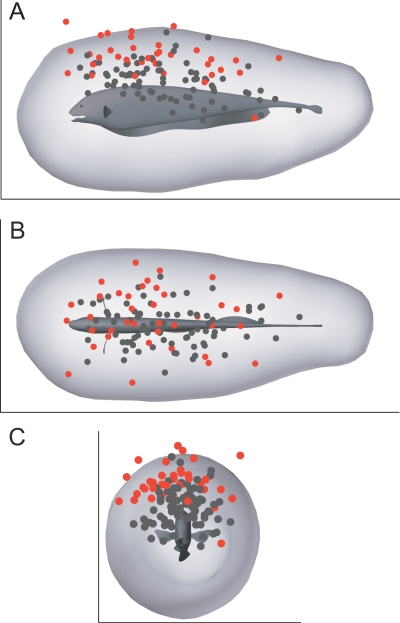
Computed Sensory Volume Overlaid with Measured Detection Locations (A) Side view. (B) Top view. (C) Front view. Dots indicate positions of water fleas (D. magna) at the time of detection (in body-fixed coordinates), based on earlier behavioral results [11]. Detections occurring at water conductivities of 100, 300, and 600 μS/cm are shown as black dots (*N* = 78), whereas detections at 35 μS/cm are shown as red dots (*N* = 38). The dorsal bias of empirically measured detection points arises from a combination of the search strategy used by the fish and the fact that prey were introduced into the tank from above [11].

We examined the active sensory volume in a weakly electric fish, the South American black ghost knifefish (Apteronotus albifrons), which captures prey in the dark using its active electric sense [10,11]. Black ghost knifefish sense their surroundings using a weak, self-generated electric field (Figure 1A). Nearby objects that differ in electrical conductivity from the surrounding water create localized voltage perturbations across the skin that are sensed by ∼14,000 electroreceptor organs (Figure 1B). In a previous prey capture study, it was shown that A. albifrons can detect small prey (Daphnia magna) in the dark at a distance of approximately 20–30 mm [11]. The detection points in the earlier study were distributed along the entire rostrocaudal axis of the fish, primarily in a hemi-cylinder above the dorsal surface (Figure 2). This dorsal bias was, in part, related to the methodology used to introduce prey into the tank in that study. Here, we used computer modeling to simulate prey trajectories that are uniformly distributed, thus allowing us to obtain a more complete and less biased estimate of the 3-D sensory volume.

**Figure 3:**
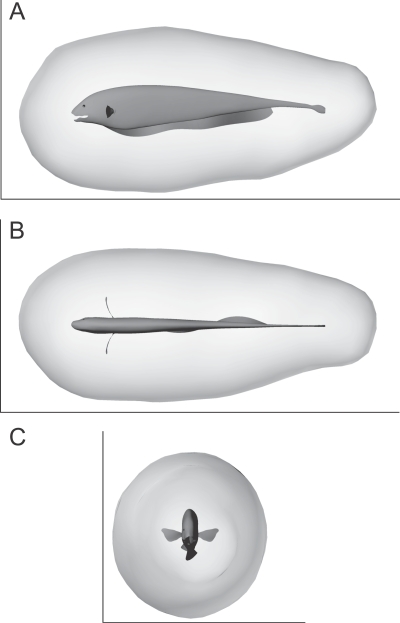
The Computationally Estimated Sensory Volume (*SV*) for Active Electrosensory Detection of D. magna by A. albifrons (A) Side view. (B) Top view. (C) Front view. Body is 14 cm long. Interactive 3-D version is available as Figure S1.

The motor capabilities of A. albifrons are as unique as its sensory capabilities. It has a multidirectional propulsion system driven primarily by a ribbon fin that runs most of the length of the body (Figure 3A; interactive 3-D version Figure S1). With this fin, in combination with pectoral fins, the fish can move forward, backward, upward, and can rapidly pitch or roll the body [11–13].

We will make comparisons between the sensory volume for prey (D. magna) and the motor volume—the locations in space that the animal can reach through activation of its musculoskeletal system. We will make these terms precise in the following section and pose hypotheses regarding possible relationships between sensory and motor volumes in animals. We will then examine these hypotheses in the context of sensory and motor volumes of electric fish.

### Definition of Motor Volume

The motor volume is the swept volume of positions a body occupies for a given trajectory or set of trajectories. It is a function of the way the body moves, as well as the geometric extent of the body. To define it more clearly, we first present a definition of the time-limited reachable set. We then use this definition to informally define the motor volume and the stopping motor volume (the swept volume of the body over trajectories that bring the body to a halt), with the precise definitions presented in Text S1.

For simplicity of description, we treat an organism as a rigid 3-D body. We define the configuration space as the six-dimensional space representing the rigid-body degrees of freedom (typically the (*x*, *y*, *z*) position of the center of mass, and θ, φ, and Ω, the yaw, pitch, and roll angles, respectively). The state space *X* consists of the six configuration components and their time rates of change (*v_x_*, *v_y_*, *v_z_*, *v*_θ_, *v*_φ_, *v*_Ω_), resulting in a total of twelve dimensions. The dynamics of the system are given by

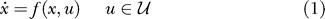
where *x* is the instantaneous state and *u* is the instantaneous control input from the space U℘ of all feasible instantaneous control inputs. The time-limited reachable set *R*(*x*_0_,*T*) is a construct from nonlinear control system theory [14–16] referring to all points in the state space *X* that can be reached by a system of the form given by Equation 1 from an initial state *x*_0_ given any feasible control history *u* of duration *T*. A feasible control history is a control input to the system as a function of time, such as muscle activations for a musculoskeletal system or steering wheel angles for a car. The reachable set is defined as:

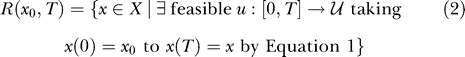



This is the set of states reachable in time exactly *T*. In our subsequent definitions, we will use the union of all reachable sets from *t* = 0 to *t* = *T*, denoted as:

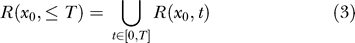



**Figure 4:**
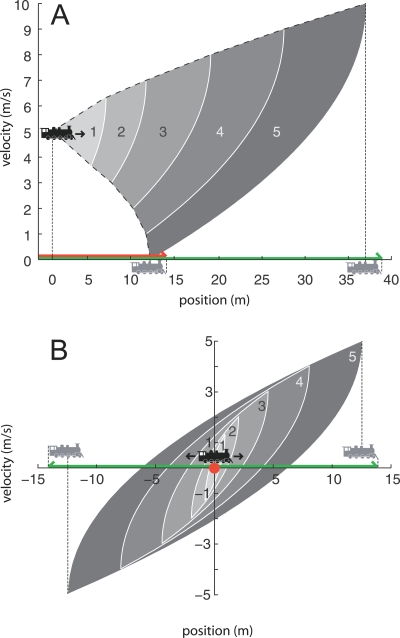
Phase Planes Illustrating the Time-Limited Reachable Set, Time-Limited Motor Volume, and Stopping Motor Volume for a Locomotive Moving on a Train Track from Two Initial Velocities Filled sectors and dashed curves refer to states for the locomotive's center of mass. The red line/dot indicates the stopping volume for the locomotive; the green line indicates the time-limited motor volume for *t* ≤ 5 s. Both are inclusive of the locomotive surface as indicated by the gray locomotives. (A) *x*_0_= (0 m, 5 m/s). Upper and lower curved dashed lines represent constant acceleration (+1 m/s^2^) and deceleration (−1 m/s^2^). Overlapping filled sectors represent reachable sets for time up to and including the labeled times, in seconds. (B) *x*_0_ = (0 m, 0 m/s).

To illustrate the concept of the time-limited reachable set, consider the simple one-dimensional case of a locomotive moving along a train track (Figure 4A). The state space *X* is simply the locomotive's configuration space (the *x* position of its center of mass), as well as the time rate of change of this one configuration component (*v_x_*). The initial condition *x*_0_= (0 m, 5 m/s). The control inputs are limited to the set U℘ = [–1 m/s^2^, +1 m/s^2^]. Figure 4A shows two dashed curves representing state space (*x*, *v_x_*) trajectories under constant acceleration (+1 m/s^2^) and deceleration (−1 m/s^2^), starting from an initial velocity of 5 m/s and running for 5 s; all time-limited reachable sets of 5 s or less from this initial condition must be within these two curves. For example, for *t* = 3 s, the time-limited reachable set *R* [(0 m, 5 m/s), ≤ 3 s] is the union of sectors “1” through “3.” Figure 4B shows time-limited reachable sets *R* [(0 m, 0 m/s), ≤ 5 s] —identical conditions as for Figure 4A but with zero initial velocity. For organisms with internal degrees of freedom (multi–rigid-body systems with joints or flexible bodies), the same concepts apply but now the state space needs to include the additional degrees of freedom, and their time rates of change.

The motor volume (Text S1, Equation S1) is similar to the reachable set but with several important differences. First, rather than a set of points in state space, the motor volume is the volume defined by the set of (*x*, *y*, *z*) coordinates of all positions occupied by the body over the time interval of interest. Second, these points are in a coordinate frame that is aligned to the body at the onset of the behavior for which the motor volume is being constructed. For the locomotive example, the thick green line between *x* = −2 and *x* = 38 in Figure 4A represents the motor volume (more correctly, the motor “line” in this case) *MV* (5 m/s, ≤ 5 s). As defined in Text S1, the motor volume is derived from the time-limited reachable set, where this set is computed from Equations 1 and 3 [17,18]. However, in our case, we do not have access to either the equation of motion (Equation 1) for the fish or to the set of feasible control histories; thus, we will estimate the motor volume empirically by examining motion capture data collected from over 100 prey capture sequences (see Methods). In this paper, we will consider the motor volume of the fish over all initial velocities that are typical within our prey capture sequence dataset. Prey capture sequences were selected to begin about 0.5 s before the onset of behavioral response and end at prey engulfment [11], and thus contain both pre- and post-detection behavior.

The stopping motor volume (Text S1, Equation S2) designates the set of all (*x*, *y*, *z*) coordinates of all positions occupied by the body from a given initial velocity to the location at which the body can be halted in the shortest amount of time. For the locomotive example, *MV*_stop_ (5 m/s) is equal to *MV* (5 m/s, ≤ 5 s) with control inputs limited to the set U℘ = [–1 m/s^2^], and is shown by the red line in Figure 4A. If the train had started at rest, then *MV*_stop_ would collapse to a single point as illustrated by the red dot in Figure 4B. In this paper, *MV*_stop_ will be ascertained through analysis of motion capture data (see Methods); if a mechanical model exists, *MV*_stop_ can be estimated by computing optimal stopping trajectories from each initial velocity.

### Definition of Sensory Volume

The sensory volume (*SV*) for a given object is the volume defined by the set of (*x*, *y*, *z*) coordinates at which that object can be reliably detected, in body-fixed or sensory system–fixed coordinates. The *SV* depends on a number of factors, such as target size, orientation, velocity, properties of the sensory system, properties of the detection algorithm, and so on. In this paper, we will estimate *SV*(prey) for the active electrosensory detection of small water fleas (D. magna).

### How Is the Sensory Volume Related to the Stopping Motor Volume?

**Figure 5:**
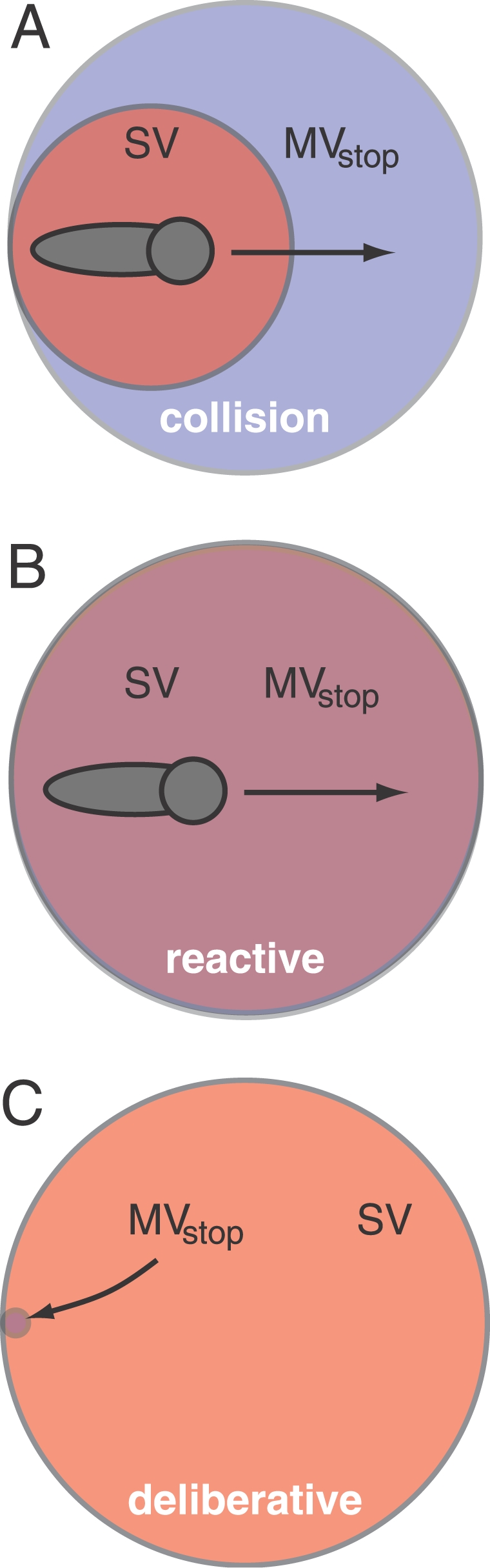
Sensory and Stopping Motor Volume Geometries (A) Collision mode: sensory volume (red) for the animal (gray) is smaller than the stopping motor volume (blue). (B) Reactive mode: sensory volume and stopping volume are equal. (C) Deliberative mode: sensory volume is much larger than the stopping volume.

With these definitions in hand, we can explore possible functional relationships between sensory and motor volumes. Restricting our consideration to the stopping motor volume, consider Figure 5, which shows three possible relationships between sensory and stopping volume geometries. In Figure 5A (“collision mode”), the sensory volume is smaller than the stopping volume. We hypothesize that this situation should rarely occur in nature when the *SV* is for objects that the animal needs to avoid colliding with. Anywhere that *MV*_stop_ is not covered by the *SV* represents a region in which unintended collisions could occur.

Figure 5B (“reactive mode”) illustrates a situation in which the *SV* and *MV*_stop_ are fully overlapping. We hypothesize that this condition represents a functional lower bound on the size and shape of the *SV*, when the *SV* is computed for obstacles that the animal should avoid. Also, if the animal's prey capture strategy demands that it come to full stop to consume or “handle” the prey, then the reactive mode can be considered a lower bound on the size and shape of the *SV* for prey capture.

Finally, Figure 5C (“deliberative mode”) illustrates the case in which the *SV* is much larger than, and fully encloses *MV*_stop_. This case is typical for visually guided predators in terrestrial environments hunting in full daylight, where visual range can far exceed stopping distance. In the context of foraging behavior, the deliberative mode would generally lead to higher foraging efficiency. However, for active-sensing systems, the sensing range could be limited by factors such as energetic costs, clutter, and quartic attenuation, among others, leading to a situation closer to the reactive mode (Figure 5B) than the deliberative mode (Figure 5C). If these constraining factors are particularly severe, then we predict that the *SV* should approximate *MV*_stop_, as in the reactive mode. Finally, we hypothesize that if the *SV* and *MV*_stop_ approximately match, then the animal may make behavioral adjustments to either the sensory volume (e.g., by changing the probe intensity) or to the stopping volume (e.g., by changing their “cruising” velocity) to maintain a matched relationship between the *SV* and the *MV*_stop_, particularly where the absence of such adjustments would bring the animal into collision mode.

Here we examine *SV–MV* relationships in the context of prey capture behavior of the black ghost knifefish. From earlier behavioral studies, we know that the fish comes to a near halt before engulfing prey [11]. Thus, we predict that *SV*(prey) should fully enclose *MV*_stop_ over the velocities that characterize pre-detection swimming. Our results will show that the relationship between *SV*(prey) and *MV*_stop_ for the black ghost is best described by the reactive mode shown in Figure 5B. For this mode, we predict that the animal might use behavioral adjustments to maintain a match between *SV* and *MV*_stop_. We test this hypothesis by reanalyzing results of our prior work with prey capture behavior in water of different electrical conductivities, which influences the distance at which prey can be detected [11]. The evidence suggests that as the *SV*(prey) shrinks in size due to increased water conductivity, the fish decreases its mean prey search velocity, which causes a corresponding decrease in the size of *MV*_stop_.

## Results

### An Omnidirectional Sensory Volume

Using synthetic prey capture trajectories, the sensory volume for active electrosensory prey detection in A. albifrons was estimated by computing a detection isosurface surrounding the fish, such that every point on the surface generates a threshold level of activation after summing and filtering the electrosensory afferent signals (see Methods). Every point contained within this bounding isosurface would yield a suprathreshold signal. The estimated *SV* was found to be omnidirectional (Figure 3), extending in all directions from the body surface. On average, the sensory surface was 34 ± 5 mm (*N =* 7,056 detection points, all numbers quoted as mean ± standard deviation unless otherwise noted) from the fish's body surface. As described in more detail below, this is also consistent with the empirically determined *Daphnia* detection distance of 28 ± 8 mm (*N* = 38) reported in an earlier behavioral study [19] once the sensorimotor delay time is factored in.

In addition to using synthetically generated linear prey capture trajectories, we also tested detection performance using empirically measured fish and prey trajectories from a previous study, which have more complex spatiotemporal profiles [11]. The computationally estimated detection distance was 33 ± 7 mm (*N* = 38 trials × 10 repeats, or 380, see Methods), compared with the measured detection distance of 28 ± 8 mm (*N* = 38 trials). However, the latter empirical value is actually the “reactive distance” at which a motor response is first observed and does not include the sensorimotor delay between detection and movement. The estimated sensorimotor delay for behavioral reactions in the knifefish is 115 ms (see Methods). Incorporating the sensorimotor delay, we obtain an estimate for the “neural” detection distance in the empirical data of 35 ± 9 mm (*N* = 38), which is in good agreement with the simulation result of 33 ± 7 mm for these trajectories.

### An Omnidirectional Motor Volume

**Figure 6:**
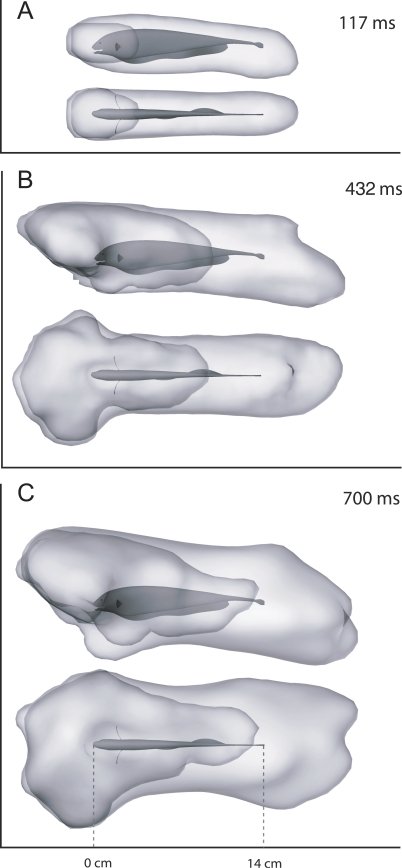
Time-Limited Motor Volumes of A. albifrons The motor volumes are shown for both the entire electrosensory array *MV*_body_ and for the mouth alone *MV*_mouth_ for three time intervals. The darker gray mouth volume is fully contained within the lighter gray body volume. (A) Side and top views at 117 ms. (B) 432 ms. (C) 700 ms. Interactive 3-D version for (A), (B), and (C) are available as Figures S2, S3, and S5, respectively.

We examined the motor volume as defined above for the entire body, *MV*_body_ as well as the motor volume for the mouth alone, *MV*_mouth_, at fourteen discrete times, ranging from 117 to 700 ms. Motor volumes at three of these time points are illustrated in Figure 6. The motor volume that maximally overlaps the *SV* (*t* = 432 ms) is shown in interactive 3-D Figure S4 along with the SV. Because of the unusually high maneuverability of the fish, including its backward-swimming capability, the shape of the body motor volume is omnidirectional and approximately cylindrical on short time scales, extending both in front of the head and behind the tail of the fish. The size and shape *MV*_mouth_, while also omnidirectional, is more compact in the rostrocaudal direction.

### Sensory Volume Encloses Stopping Motor Volume

**Figure 7:**
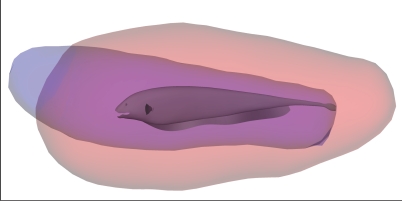
The Measured Stopping Motor Volume for A. albifrons (Blue) and the Computed D. magna Sensory Volume (Red) Interactive 3-D version is available as Figure S6.

Figure 7 shows the relationship between the *SV* and the stopping motor volume. The *SV* fully encloses *MV*_stop_ except for a rostral protrusion.

## Discussion

To interpret the sensory and motor volumes presented in this study, an analogy may prove useful. When driving an automobile at night, the driver's visual system combines with the automobile's headlights to form an active visual sensing system. Convolving the sensory coverage and sensitivity of the human visual system with the angular coverage and intensity profile of the headlights would permit a sensory volume to be estimated for night driving. The stopping volume, in this case, is the set of end points of minimal-time trajectories which bring the vehicle to a stop. This volume depends on the set of initial velocities, roadway conditions, braking power, and reaction time of the driver, among other factors. Having constructed sensory and stopping motor volumes for the night driving example, it would then be useful to determine whether a driver would be in collision, reactive, or deliberative mode (Figure 5) with respect to a pedestrian that comes into view. A lower bound on the stopping volume, for example, would be *MV*(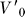 ≤ *RT*), where 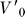 is the cruising velocity (Equation S1) and *RT* is the driver's reaction time, under certain assumptions about braking power, road conditions, etc.

### Omnidirectional Motor Capabilities

In the present study, we find that the time-limited motor volumes for A. albifrons are omnidirectional and extend equally in front and behind the fish (Figure 6; interactive 3-D version Figures S2, S3, and S5). This confirms the previously reported propensity of weakly electric knifefish to spend a significant fraction of time swimming backward at velocities comparable to their forward-swimming velocities [13]. The motor volume is found to be cylindrical, indicating that the fish has considerable lateral and dorsoventral mobility [11,12]. The time-limited motor volume introduced in this study provides a quantitative measure of the fish's motor capabilities as a function of time interval.

The body motor volume was closer in shape to the sensory volume and exhibited greater overlap than the mouth motor volume (Figure 6). Functionally, the mouth motor volume is more closely associated with prey capture behavior, whereas the body volume may be more relevant to obstacle avoidance and general navigation in complex environments. The sensory volume seems better matched to the body motor volume, which suggests that general navigational capabilities, habitat complexity, and obstacle avoidance should all be considered when examining relationships between sensory and motor volumes in the context of prey capture behavior.

### Omnidirectional Sensing

We find that the active sensory volume for prey detection is also omnidirectional and approximately cylindrical (Figure 3). The omnidirectional shape of the *SV* is similar to the shape of the isopotential surfaces of the self-generated electric field surrounding the fish (Figure 1A), but exhibits less of a bulge in the caudal tail region due to anisotropies in the sensor density (Figure 1B), field intensity (Figure 1A), reduced sensory surface area due to the tapering body morphology (Figure 1B), and the sensitivity of the primary electrosensory afferents to prey-induced perturbations [20,21]. The relative importance of these factors in determining the precise shape of the sensory volume was not examined. It will be particularly interesting in future studies to explore the extent to which the higher electroreceptor density in the head region of the fish influences prey-detection distance versus spatial resolution of prey position. The simulations of prey detection in this study combined quantitative models of each of these factors in order to arrive at an estimated sensory volume for electrosensory-mediated prey detection. As shown in Figure 2, there was good agreement between the computationally estimated sensory volume and the empirical distribution of prey detections found in an earlier study [11]. The empirical study had relatively low *N* (38 prey capture events for the water conductivity that yielded maximum detection range), and the detection points were biased toward the dorsal surface of the fish, because prey were introduced into the tank from above. The computational approach used here allowed us to obtain a more complete and less biased estimate of the fish's sensory detection volume.

### Enclosure of the Stopping Motor Volume by the Sensory Volume

Our prediction that the fish avoids collision mode is supported by Figure 7 (interactive 3-D version Figure S6), which shows nearly full enclosure of the stopping volume by the sensory volume. The stopping volume was taken from just before detection (which always occurred during forward movement) to the point of zero forward velocity as the fish reverses to capture the prey. Thus, unlike the time-limited motor volume, which sampled all initial velocities including negative velocities, the stopping volume is more strongly biased in the forward direction.

However, the extent of the sensing range is restricted to nearly the lower limit of the reactive mode (Figure 5B) where the *SV* and *MV* are matched. We expect this is due to constraints that include the metabolic cost for emitting energy into the environment and the interference caused by interaction between the emitted signal and nearby clutter. Energetic costs scale steeply with sensing range, approximately as a quartic power of the range due to geometric spreading effects on the outbound and return paths of active probe signal [1,3]. For quartic scaling, doubling the active-sensing range would require a 16-fold increase in emitted energy. To appreciate the effect of this scaling, consider that an active 15-g A. albifrons requires approximately 300 J/day [22]. If we assume that ≈1% is used for the field, as estimated for another weakly electric fish [23], then 3 J/day is needed for the field. To double the detection distance for D. magna from the measured ≈30 mm to ≈60 mm would therefore require 48 J/day for the field; to double this again to ≈120 mm (still less than one body length for a 15-g fish) would then require 768 J/day, more than double the entire energy budget of the fish. Thus, the high energetic costs associated with extending the active-sensing range is likely to place strong selective pressure on the shape and extent of the active sensory volume. In comparison, the high acuity passive visual system of a typical raptor allows it to spot prey from over a kilometer away, or about 10,000 body lengths.

Although it is more difficult to provide a quantitative metric for the interference effects from clutter, the great reduction in emission power observed in dolphins and bats when surrounded by clutter or when nearing a target (see Introduction) suggests that this may also be an important factor in limiting the desirable range of an emitted signal. Returning to the automobile scenario, driving at night in a dense fog provides a practical example of where backscatter is a limiting constraint. Increasing headlight intensity under these conditions (e.g., switching from low beams to high beams) can actually degrade detection performance because the “noise” from backscattered light makes it more difficult to detect the “signal” that is reflected back from a target of interest.

Given that the black ghost is in reactive mode, we predict that the fish may make behavioral adjustments to either the sensory volume or to the stopping volume to maintain a matched relationship between the *SV* and the *MV*_stop_, particularly where the absence of such adjustments would bring the animal into collision mode. We are able to qualitatively evaluate this hypothesis by examining how search (predetection) swimming velocity varies with water conductivity. We have shown that water conductivity changes the range at which prey are detected [11]. In the previous study [11], we found that the mean detection distance decreased from 28 mm at a water conductivity of 35 μS/cm to 12 mm at 600 μS/cm. Over this conductivity range, the fish's predetection swimming velocity decreased 30% from 99 mm/s to 71 mm/s. At the shorter detection distances associated with higher conductivity water (600 μS/cm), we have previously estimated that multiple sensory modalities, including passive electrosense and lateral line mechanosense, are playing a role [24]. Thus, quantitative evaluation of the matching hypothesis would require modeling the *SV* for these other sensory modalities, which is outside the scope of this study.

### Neural Implications

The size of the estimated sensory volume, and to a lesser degree the shape of the volume, are influenced by the properties of the neural detection algorithm. A more sensitive algorithm will result in a larger detection range. The detection algorithm used here is not intended to model the fish's actual detection performance in detail. Doing so would require a more extensive analysis of additional factors, such as sensory reafference associated with tail bending, environmental background noise, contributions of other sensory modalities, neuroanatomical constraints on sensory convergence, etc. Rather, the detection volume reconstructed here is intended as an estimate of “best-case” detection performance based solely on changes in active electrosensory afferent spike activity.

### Comparison with Other Active-sensing Species

Echolocating bats emit ultrasonic energy into the environment to detect prey [25]. While the precise size and shape of the bat's sensory volume will vary with many factors (species, call intensity, duration, etc.), the sensory volume for echolocation is generally a cone that extends in front of the head of the bat with an angular range of approximately ±30° in azimuth and elevation [26]. The angular coverage may extend as much as ±75° relative to the body axis when head and pinnae movements are included [25,27,28]. For the detection of flying insects by pipistrelle bats, the sensory volume extends at least 100–200 cm in front of the animal [25,29] based on the reactive distance to prey.

The detailed shape of the bat's motor volume has not been reported. The stopping distance can be estimated by combining information on the initial velocity of the bat, maximal deceleration, and sensorimotor time delay. Taking a representative bat cruising velocity of 5 m/s [25], a maximal deceleration of 15 m/s^2^ (estimated from a sample trajectory in [25]), and an estimated sensorimotor delay of 100–200 ms [30] yields an estimated stopping distance in the range of 130–180 cm. Although there is a great deal of uncertainty in these estimates, the stopping distance of the bat seems comparable to the sensory range for prey detection. This suggests that bats, like electric fish, have an active sensory volume for prey detection that may be comparable to their stopping volume. Quantitative comparisons of sensory and motor volumes for a single bat species would help clarify these relationships.

Odontocetes (toothed whales, dolphins, and porpoises) also use ultrasonic energy for prey detection. Dolphins can detect prey-sized objects at distances on the order of 100 m [6,31]. The 100-m sensing range of dolphins is certain to be significantly beyond their stopping volume, although there is little published data with which to make quantitative comparisons. This suggests that energetic and clutter-related constraints on active sensing may not be as significant for dolphins as they are for bats and electric fish.

### Comparison with a Passive-sensing Species

**Figure 8:**
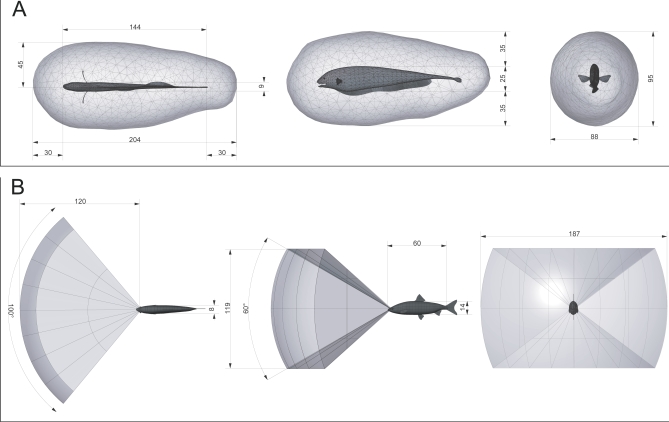
Comparison of Active and Passive Sensory Volumes for Prey Detection in Fish, Dimensions Shown in Millimeters (A) Omnidirectional sensory volume (from Figure 3) for electrosensory prey detection for black ghost knifefish (A. albifrons, length 144 mm) detecting water fleas (D. magna, length 2–3 mm). The angular coverage is omnidirectional and the range is 34 mm on average. (B) Forward-directed sensory volume for passive vision, based on prey reaction volumes for stone moroko (Pseudorasbora parva, length 60 mm) feeding on water fleas (*D. pulex,* length 1–2 mm). The angular coverage is approximately 100° in azimuth and 60° in elevation, with a range that varies from about 60–120 mm, depending on environmental conditions [36]; 120 mm is shown here. The two volumes are similar in size (1,000 cm^3^ each) but quite different in shape. Interactive 3-D version is available as Figure S7.

Both the active electrosensory volume for prey detection and the time-limited motor volume of A. albifrons are omnidirectional and approximately cylindrical. This is in striking contrast to most other animals, which tend to exhibit a strong forward bias in both sensory and motor volumes. This forward bias is observed for passive-sensing systems such as visually guided fish [32–37]. Figure 8 (interactive 3-D version Figure S7) compares the omnidirectional prey sensory volume in the black ghost knifefish (A. albifrons) with a more typical forward-biased passive sensory volume. The latter is illustrated by the volume for visually mediated prey detection in the stone moroko (Pseudorasbora parva), a fish of comparable size that also feeds on *Daphnia* [36]. The angular coverage of the visual volume is approximately 100° in azimuth and 60° in elevation, with a range that varies from about 60–120 mm, depending on environmental conditions [36]; the 120-mm range is shown in Figure 8. The active electrosensory volume of A. albifrons and the passive visual volume of P. parva for prey detection are similar in size (approximately 1,000 cm^3^ each) but quite different in shape.

### A New Metric for Control System Analysis

We propose that the ratio of the sensory volume to stopping volume (*SV*:*MV*_stop_) is a useful metric for both active and passive sensory systems when considering whether sensorimotor control systems are in collision, reactive, or deliberative mode. Collision mode occurs when the ratio is below unity. Reactive mode occurs when the ratio approximates unity, as appears to be the case for knifefish and bats. In this mode, sensorimotor control algorithms are likely to be reactive, with relatively fast, direct coupling between sensation and action. Movement options are largely conditioned by mechanical considerations such as inertia and minimal turning radius. For example, some sensor-based motion planning algorithms in robotics are based on estimating the stopping volume for the nearest obstacle; as the robot becomes more massive, the range of any active-sensing system for obstacle detection must be extended accordingly [38]. Deliberative mode occurs when the ratio is large, as for dolphin echolocation and for many passive visual and auditory systems. In this mode, an animal can acquire sensory data from targets that are far outside its stopping volume. This allows the animal a greater range of movement options, because there is adequate time for complex motion planning before reaching the target [39]. For example, in the context of prey capture behavior, a dolphin with a high *SV*:*MV*_stop_ ratio is able to engage in long-range tracking of distant prey, whereas a weakly electric fish with a ratio near unity must use a reactive strategy for chance encounters with nearby prey. Quantitative analyses of sensory and motor volumes for both active and passive-sensing systems can highlight important functional relationships between sensing, movement, and behavioral control in animals.

## Materials and Methods

### Behavioral data.

In a previously published study [11], adult weakly electric fish (A. albifrons) were videotaped in a light-tight enclosure under infrared illumination. Individual water fleas (D. magna, 2–3 mm in length) were introduced near the water surface and drifted downward; prey capture behavior was recorded using a pair of video cameras oriented along orthogonal axes. Relative to the fish's velocity (∼100 mm/s) the prey were relatively stationary (prey velocity < 20 mm/s). Prey capture events (from shortly before detection to capture) were subsequently digitized, and 3-D motion trajectories of the fish surface and prey were obtained using a model-based tracking system with a spatial resolution of 0.5 mm and a temporal resolution of 1/60 s [40]. The time of prey detection was defined by the onset of an abrupt longitudinal deceleration as the fish reversed swimming direction to capture the prey [11]. These reversals are characteristic of most prey capture encounters. This is related to the fact that the fish tends to swim forward with its head pitched downward, such that the dorsum forms the leading edge as the fish moves through the water. Initial prey encounters thus tend to be uniformly distributed along the entire length of the body, so a reversal of swimming direction is typically required to intercept the prey.

### Sensory volume (*SV*) for electrosensory prey detection.

The volume of space supporting prey detection by the active electric sense was estimated computationally using measurements and empirically constrained models of the prey, electric field, fish body and sensor distribution, electrosensory images, afferent firing dynamics, and behavior. Model parameter values and their sources are summarized in Table 1.

**Table 1:**
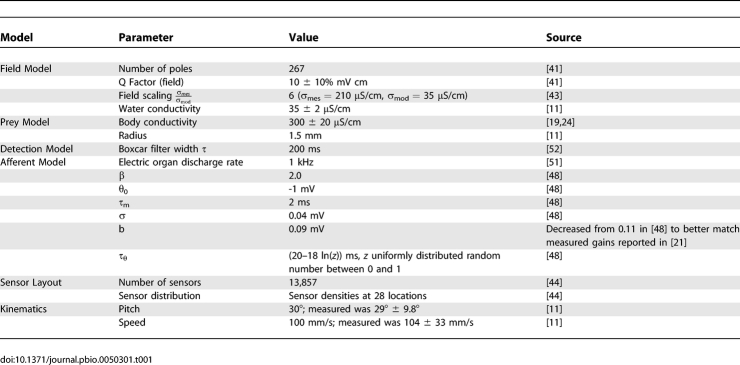
Model Parameters

Electric field. The electric field vector **E_fish_** (mV/cm) at a 3-D point in space **x** was computed using an empirically tested model of the electric field [41]. This model sums the individual contributions to the field from each of a series of charged poles used to model the electric organ of the fish:

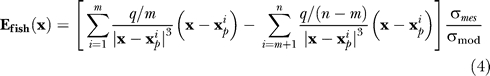
where **x** is a point in space (cm), 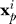 is the location of pole *i* of *n* total poles, *q* is a normalization constant (mV cm) that scales the overall magnitude of the field, σ_mes_ is the conductivity of the water that the field measurements were performed in (which establishes the *q* value), and σ_mod_ is the conductivity of the water for the simulated field. The quantity *q* is analogous to electric charge in an electrostatic model and is distributed such that the first *m* poles have a “charge” of *q/m* and the remaining poles have a charge of *–q/*(*n – m*), resulting in a total net charge of zero. For our simulations, *n* = 267, *m* = 266 (all but one pole at the tail was positive), *q* = 10, and the pole locations **x**_p_ ran from the nose to the tail of the fish along the central axis of the fish body with equidistant spacing. These values resulted in field values within 10% of measurements of the electric field vector **E_fish_** of A. albifrons obtained by other researchers (B. Rasnow, C. Assad, P. Stoddard, unpublished data) in water of conductivity σ_mes_ = 210 μS/cm using a multiaxis electrode array [20,42] (Figure 1A). The 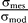 term scales the field strength to the water conductivity used in simulation σ_mod_ = 35 μS/cm. This scaling is based on empirical measurements of the knifefish field at different water conductivities [43], which suggest the electric organ can be idealized as constant current source. We selected 35 μS/cm because our earlier study [11] found that the detection range was highest for trials at this conductivity, and this conductivity is most similar to rivers of the fish's native habitat.

*Body model with electroreceptor distribution.* We used a prior survey [44] of the density of probability type (P-type) tuberous electroreceptor organs (hereafter electroreceptors) on the surface of A. albifrons. These are the dominant electroreceptor type for A. albifrons [45,46]. Each electroreceptor is connected uniquely to a primary afferent, which generates action potentials with a probability that varies with stimulus intensity. The receptor locations were mapped onto a high-resolution polygonal model of the fish derived from a 3-D scan of a body cast [19,24] in accordance with the measured sensor densities [44] (Figure 1B). This resulted in a total of 13,857 mapped electroreceptors, in close agreement with neuroanatomically derived counts from A. albifrons [44].

Prey model. Based on prior measurements of live prey (*D. magna)*, it was modeled as a 1.5-mm-radius conductive sphere with an electrical conductance of σ_obj_ = 300 μS/cm [19,24]. Idealizing the prey as a sphere allows us to use an analytical model for the stimulus caused by the prey, described below.

*Electrosensory image formation*. The voltage perturbation Δφ (mV) at an electroreceptor on the fish surface, arising from a small spherical target object, was computed using an empirically tested model [47]:

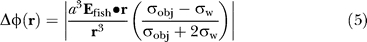
where **E_fish_** (mV/cm) is the electric field vector at the prey, **r** (cm) is the vector from the center of the spherical object to the electroreceptor on the fish surface, *a* is the radius of the sphere (cm), σ_obj_ is the conductivity of the sphere, and σ_w_ is the conductivity of the water (μS/cm). Simulations were run with water conductivity σ_w_ set to 35 μS/cm (see Methods, Electric field).

*Primary afferent spiking activity*. Computed voltage perturbations at each sensory receptor on the fish body were transformed into primary afferent spiking activity using a previously published adaptive threshold model of P-type (probability coding) primary electrosensory afferent response dynamics [48]. This model gives rise to negative correlations in the interspike interval (ISI) sequence, which lead to long-term spike train regularization. This correlation structure has been shown to increase information transfer and improve detection performance for weak signals [48–50]. The electric organ discharge (EOD) frequency was taken as 1 kHz, which is typical of A. albifrons [51]. P-type afferents fire at most one spike per EOD cycle. Thus, afferent activity was modeled as a binary spike train with a sampling period equal to the EOD period (1 ms). On each EOD cycle (*n*), the following update rules are evaluated in order:

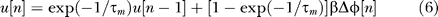


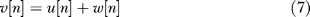


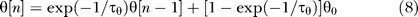


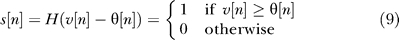


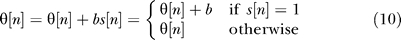



Equation 6 implements low-pass filtering of the voltage perturbation Δφ[*n*] with gain β and time constant τ_m_. The state variable *u*[*n*] can be conceptualized as a membrane potential; it is initialized to 0, corresponding to the steady-state value with no stimulus present (Δφ = 0). Equation 7 adds random noise to *u*[*n*] to create a noisy membrane potential *v*[*n*]; the noise *w*[*n*] is modeled as zero-mean Gaussian noise with variance σ^2^. The actual noise distribution is likely to be more complex, but the Gaussian approximation adequately captures available empirical data. Equation 8 describes the behavior of an adaptive spike threshold θ[*n*] that decays toward a baseline threshold θ_0_ with a time constant τ_θ_. Equation 9 represents the process of spike generation, where *s*[*n*] is the binary spike output (*s* = 1, spike; *s* = 0, no spike); *H* is the Heaviside function, defined as *H*(*x*) *=* 0 for *x <* 0 and *H*(*x*) *=* 1 for *x ≥* 0. A spike is generated whenever the noisy membrane potential *v*[*n*] exceeds the threshold θ[*n*]. Equation 10 implements a relative refractory period by elevating the threshold θ[*n*] by an amount *b* immediately following a spike. (The threshold level subsequently decays toward its steady state value according to Equation 8.)

**Figure 9:**
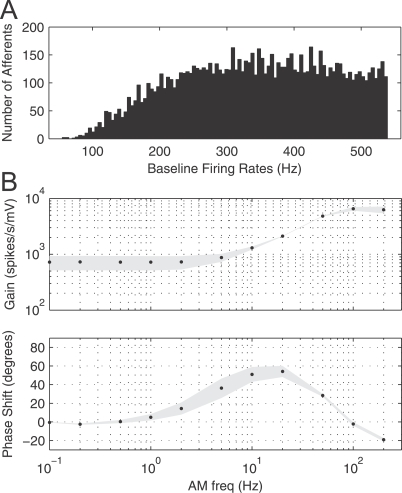
Gain, Phase Response, and Firing Rate Distribution for Afferent Model (A) Histogram displaying baseline (2 s, no stimulus) firing rate distribution for modeled afferents (*N* = 10,000). Mean firing rate is 0.34 ± 0.11 kHz, similar to 0.33 ± 0.13 kHz measured in vivo [53]. (B) Gain and phase response of modeled P-type afferents (*N* = 5) (minimal change for larger groups) for 20-s sinusoidal stimuli. Frequencies range from 0.1 to 200 Hz. Black dots indicate mean values, gray areas depict one standard deviation. Profiles are similar to [21] for simulations and experimental.

Parameter values common among all afferents in the model were β = 2.0, *b* = 0.09, σ = 0.04, θ_0_ = −1, and τ_m_ = 2 [48]. The values of the time constant τ_θ_ were generated independently for each afferent according to τ_θ_ = 21 – 18 ln(*z*), where *z* is a uniformly-distributed random number between 0 and 1. This distribution for τ_θ_ results in a distribution of baseline firing probabilities in the model that is matched to the empirically observed distribution [52] (Figure 9A). The initial value for the threshold level θ[*n*] was drawn randomly from a Gaussian distribution with mean (0.064) and standard deviation (0.045) matched to the steady-state distribution of threshold values obtained from the model with no stimulus present (Δφ = 0). As detailed in Brandman and Nelson [48], these parameter choices result in a distribution of baseline firing rates, gains, and amplitude modulation–frequency tuning properties similar to empirically measured values [21,53], as shown in Figure 9. At the time of prey detection, the peak change in transdermal voltage is on the order of 1 μV, and the peak change in afferent firing rate is on the order of 1 spike/s [12,54].

**Figure 10:**
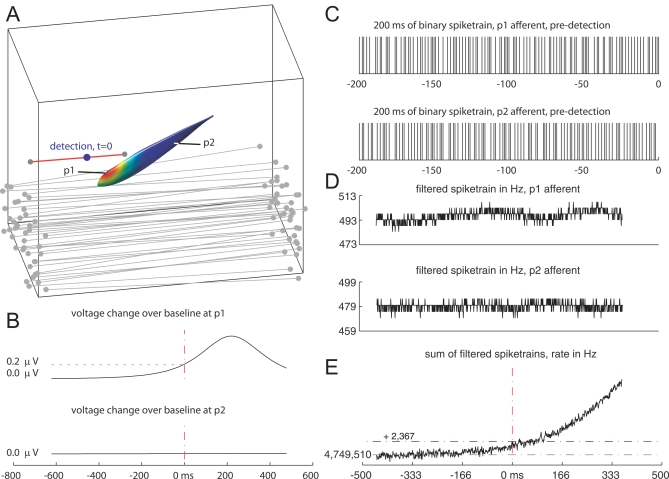
The Process of Estimating the Sensory Volume for *Daphnia* (A) First, simulated prey are moved along forward and backward rays at a speed of 100 mm/s. A subset of the 1,014 forward rays is shown in gray. The red ray indicates a trajectory where prey detection occurred (blue dot, *t* = 0). (B) The estimated change in voltage across the skin at **p1**, induced by the relative motion between the fish and prey, is shown in comparison with the voltage across the skin at **p2**, further away from the stimulus. Detection occurred in this case with a 0.2-μV change on a 1-mV baseline, a 0.02% change. (C) A short 200-ms segment of the spike trains corresponding to afferents at **p1** and **p2**. (D) The spike trains are low-pass filtered to obtain a continuous estimate of spiking rate changes relative to baseline. (E) On each 1-ms time step, the filtered activity of all afferents is summed. The summed activity is thresholded at a level that gives a 10% false alarm rate over ten repeats (one false detection); the location of the prey at the time of threshold crossing is used to define the detection point. Steps A–E are repeated for each of 1,014 forward rays and of the same number of backward rays to construct a cloud of detection points. These points are then processed to form the *SV* surface.

*Simulated prey encounter trajectories*. During the search phase of prey capture behavior observed in earlier studies, A. albifrons typically swims forward with a mean longitudinal velocity of ∼100 mm/s, with its head pitched downward at an angle of ∼30° relative to horizontal [11]. The slowly moving prey were relatively stationary, with typical velocities less than ∼20 mm/s [11]. In the current simulation study, we modeled this relative motion between the fish and prey by moving the prey target along horizontal rays at 100 mm/s toward a stationary model fish pitched downward at 30° (Figure 10A). Two sets of such prey trajectories were simulated, one set consisting of trajectories from head-to-tail, the other set from tail-to-head, since the fish swims forward and backward. A static model of the fish (140 mm long) was centered within a rectangular box at a pitch of 30°. The box size was chosen such that any prey trajectory originating on a box face would begin well outside the detection range of the fish. The shortest distance between a point on any face of the box and any point on the fish was 60 mm. This distance was set by examining preliminary simulations and empirical measurements, which showed typical detection distances of 20–40 mm. The resulting dimension of the box was 245 mm (length), 129 mm (width), and 193 mm (height). For the head-to-tail trajectories, horizontal prey positions from the front plane to the back plane were generated with starting and ending points centered on a grid with spacing of 5 mm (a total of 1,014 trajectories). Variation in starting and ending points was achieved by adding random values ranging from negative to positive one-half the grid spacing. Intervening points on the trajectory were on a straight line, at a time interval of 1/60th of a second. The same number of tail-to-head trajectories were generated in the same manner.

### Estimating the locations of prey detection from spike train activity.

For each simulated trajectory, we use a detection algorithm to estimate the point at which the prey should have become detectable based on changes in afferent spike activity. The detection points that emerge from this analysis are then used to estimate the size and shape of the electrosensory prey detection volume. The approach used here is not intended to model the fish's actual detection performance in detail. Doing so would require a more extensive analysis of additional factors, such as sensory reafference associated with tail bending, environmental background noise, contributions of other sensory modalities, neuroanatomical constraints on sensory convergence, etc. Rather, the detection volume reconstructed here is intended as an estimate of “best-case” detection performance, based solely on changes in electrosensory afferent spike activity.

The voltage perturbation corresponding to each prey position was computed from Equation 5 across the full complement of 13,857 sensory receptors. The resulting history of voltage perturbations at each receptor was interpolated to produce values at each millisecond and used as input for Equation 6. Because of the stochastic nature of the afferent model, ten spike trains were generated for each afferent voltage history for each trial. Output spike trains for these synthetic trials were individually filtered using a boxcar filter with a window size τ = 200 ms, which in previous studies has corresponded to the best weak signal detection capability [52]. At the end of each millisecond, we extract and sum the scalar activity level of each of the 13,857 afferents. The motivation for this approach is that we assume that any neural detection algorithm will require pooling of information from multiple afferents; our goal here is not to specify exactly how this pooling process takes place, but rather to evaluate a best-case scenario. Using the “no-stimulus” condition, a detection threshold was established at a level that yielded one false detection per ten repeats. This detection strategy is a population-level extension of the algorithm described by Goense and Ratnam [54] for detection of weak signals in an individual spike train with ISI correlations.

The prey detection distance was defined by the position of the prey at the time of threshold crossing for the “stimulus” condition. For each prey trajectory, the median and standard deviation of the detection distances were computed over ten repeats. The number of detections over all trajectories formed a bimodal distribution, with one peak near one, corresponding to “false alarms” for trajectories outside of the detection range, and a second peak near ten, corresponding to “true hits” for detections well within sensory range. There was a minimum in the bimodal distribution around seven, and we retained all trajectories with eight or more detections (out of ten) for further analysis. The mean and standard deviation of the detection distance for the resulting cloud of detection points was computed. To create the *SV*, this point cloud was triangulated into a smooth 3-D surface representation using a commercial software package (RapidForm, INUS Technology, Seoul, South Korea).

*Measured prey encounter trajectories*. The synthetic prey capture distance results were validated by following the same methodology outlined above using measured prey encounter trajectories from an earlier study [11]. The earlier study found best detection performance for trials in low conductivity water most similar to the water of the fish's native habitat. Therefore, we examined only this subset (35 μS/cm, *N* = 38). In our prior study, the measured detection locations were estimated by examining the first change in behavior near to the prey [11] and therefore include sensorimotor delay time. Thus, to compare these to the computed neural detection points, we retrieved the distance between the prey and the fish at the detection time minus the sensorimotor delay time (115 ms [55]).

### Motor volume (*MV*).

The estimation of *MV* from motion capture data does not assume the fish is stationary at the initial time step; rather, *MV* is estimated over all the initial velocities typical of our prey capture behavioral segments starting from just before detection to capture, which includes forward as well as backward velocities, in addition to heave (up in the body frame), and angular velocities such as roll and pitch*.* It is defined in the coordinate frame at the fish's initial position (see Text S1 for details). We consider both the 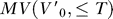 , where 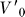 is the initial velocity in the coordinate frame fixed to the fish's initial position, for the entire body surface, *MV*_body_, as well as the 3-D motor volume for the mouth alone, *MV*_mouth_.

The *MV* was computed from the 3-D fish surface trajectory data obtained from 116 reconstructed prey-capture trials from an earlier study [11]. Because the motor volume for the mouth is a subset of this space, we will simply refer to the full volume as the *MV*, unless the distinction between mouth and body volumes is relevant. 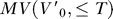 was estimated from the trajectory data at fourteen different times *T*, ranging from 117 to 700 ms. For a given *T*, the nodes on the surface of the polygonal fish model at time *t*_init_ *+ T* were transformed back into the body-centered coordinate system of the fish at time *t*_init_ . This was repeated over all possible starting times *t*_init_ for each trajectory (every 1/60th of a second up to the length of the trial minus *T*), thus uniformly sampling all observed velocities. The result of this procedure was a dense point cloud, representing where points on the fish's surface can reach over time *T*. The points on the surface of this cloud delineate an empirical estimation of 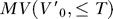 (Equation S1) in the absence of the equation of motion (Equation 1) and feasible control histories, as discussed in the Introduction. The surface of the motor volume at each of the 14 intervals was defined by binning of the point cloud around the fish into voxels (5 × 5 × 5 mm), smoothing the data with a 3-D Gaussian convolution kernel (standard deviation, 5 mm), and setting a threshold to include all voxels with point counts up to the 95th percentile. Each resulting point cloud was triangulated to form closed polyhedra for further analysis using commercial software (RapidForm, INUS Technology, Seoul, South Korea). The motor volume for the mouth was constructed following the same procedure as for the body, but using only a single node at the rostrum of the polygonal body model. Because only a single node was used, the resulting point cloud was less dense than the body point cloud. To accommodate the lower density, we maintained all voxels with point counts up to the 90th percentile (rather than the 95th percentile used for the body) when constructing *MV*_mouth_.

The stopping volume was constructed similarly, but for comparison of the stopping volume to the *SV*, we restrict our selection of trials to those of the same conductivity as used for estimating the *SV*, a total of 38 trials at 35 μS/cm. Unlike the *MV*, *MV*_stop_ is not a function of a fixed time *T* but rather depends on the set of initial velocities 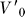 from which we monitor movement until zero velocity is reached (see Text S1). Thus, we examine the volume swept by the body from the behavioral reaction (detection) time minus the sensorimotor delay time (115 ms, [55]) to the time at which the longitudinal velocity of the body is zero. We take the union of these 38 volumes to derive the stopping volume over all 35 μS/cm trials.

### Computing environment.

Computations were performed on a 54 CPU (2 GHz G5, 1 GB RAM) cluster of Xserves (Apple Computer, Cupertino, California, USA) running OS X. An open-source distributed computing engine (Grid Engine, Sun Microsystems, Santa Clara, California, USA) was used to manage the computation across the nodes. Simulation and analysis programs were written in MATLAB (The Mathworks, Nantick, Massachusetts, USA) and compiled to portable executables for execution on the cluster.

## Supporting Information

Interactive 3D visualizations of the sensory volume for prey (water fleas, D. magna) (*SV*), time-limited motor volume (*MV*), and stopping motor volume (*MV_stop_*) for A. albifrons, the black ghost knifefish.

These Virtual Reality Markup Language (VRML) models can be viewed using downloadable web browser plugins and external viewers available for many platforms.

As of the date of publication, one of the following is recommended, in order of preference:

Cortona VRML plugin (Windows and Mac OS X; available at: http://www.parallelgraphics.com/products/cortona).

Octaga VRML player (Windows, Mac OS X. and Linux; available at: http://www.octaga.com/).

Xj3D viewer (Windows, Mac OS X. and Linux; available at: http://www.web3d.org/x3d/xj3d/).

Figure S1 Interactive 3-D Version of Figure 3
(1.3 MB VRML)

Figure S2 Interactive 3-D Version of Figure 6A (1.3 MB VRML)

Figure S3 Interactive 3-D Version of Figure 6B (1.7 MB VRML)

Figure S4Sensory Volume (Red) and Time-Limited Motor Volume 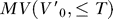 (Blue) at the Time of Maximal Overlap between SV and MV (*t* = 432 ms)
(1.6 MB VRML)

Figure S5 Interactive 3-D Version of Figure 6C (1.6 MB VRML)

Figure S6 Interactive 3-D Version of Figure 7
(1.4 MB VRML)

Figure S7 Interactive 3-D Version of Figure 8
(1.8 MB VRML)

Text S1 Technical Definition of Motor Volume and Stopping Motor Volume

## Abbreviations

EOD: electric organ discharge
*MV*: time-limited motor volume
*MV*_body_: time-limited motor volume for the body
*MV*_mouth_: time-limited motor volume for the mouth
*MV*_stop_: stopping motor volume
P-type: probability-type primary afferent
*SV*: sensory volume
