# Supplementary material for: Omnidirectional Sensory and Motor Volumes in Electric Fish
Source: PLoS Biol. 2007 Nov 13;5(11):e301. doi: 10.1371/journal.pbio.0050301 (PMC2071945; doi:10.1371/journal.pbio.0050301)
Supplement: Supplementary file 1 [file pbio.0050301.sd001.pdf]

TEXT S1: Definition of the Motor Volume and Stopping Motor Volume

We can define $R'(x_0, T)$ to be the set of reachable states expressed in the world (inertial) frame coincident with the body's frame at time $t = 0$ (call this frame B). This set is just a coordinate transformation away from the set $R(x_0, T)$ (Eq. 3). We will refer to states, expressed in the frame B, as elements of the state space X'. On the assumption that the reachable set, expressed in B, does not depend on the initial configuration in world coordinates, we can then eliminate the configuration portion of x_0, giving $R'(v_0', T)$, where v_0' is the velocity expressed in B. An example of where this assumption does not hold is in certain cases of a body in gravity. For example, the reachable set for a locomotive on a track of varying slope depends on its position on the track: the train on a steep track will have a different (relative) reachable set than the train on a horizontal track. On the other hand, this assumption holds approximately for the knifefish under study. Within certain limits, the dynamics of the fish are independent of its *x,y,z* location in water, and because gravity is opposed by swim bladder buoyancy, the orientation of the fish has minimal effect on its reachable sets when expressed in the frame B.

For a state χ of a body in the reachable set $R'(v_0', T)$, we define $\text{Proj}(\chi)$ as the projection of χ from the twelve-dimensional space X' to the six-dimensional configuration space expressed in the frame B. $\text{Vol}(\text{Proj}(\chi))$ is a function which returns the set of points (3D coordinates in the frame B) occupied by the body at $\text{Proj}(\chi)$.

These definitions allow us to define the motor volume as $MV(v_0', T) = \bigcup_{\chi \in R'(v_0', T)} \mathrm{Vol}(\mathrm{Proj}(\chi))$,

from which we can define the motor volume for $0 \le t \le T$ as

$MV(v_0', \le T) = \bigcup_{t \in [0,T]} MV(v_0', t)$. Finally, we can generalize this over a set of initial

velocities expressed in the frame B in the set V_0', as we will use in this paper:

$$MV(V_0', \le T) = \bigcup_{v_0' \in V_0'} MV(v_0', \le \mathrm{T}) \text{ (Equation S1)}$$

A volume related to the *MV* is the *stopping* motor volume, MV_{stop}. MV_{stop} is related to the *MV*, but for each initial velocity v_0', we determine a trajectory that brings the body to a halt with a feasible control history *u* under some criterion such as minimum time or minimum distance. This trajectory is determined by a *stopping controller* $SC(\cdot)$ that takes an initial body velocity v_0' and returns an entire feasible trajectory (including the total time of motion t_f) $\chi : [0, t_f] \rightarrow X'$ with the property that the body is halted at time t_f. Then the stopping volume for an initial velocity v_0' is written

$MV_{stop}(v_0') = \mathrm{Vol}(\mathrm{Proj}(SC(v_0')))$. Finally, for a set V_0' of initial velocities, we have

$$MV_{stop}(V_0') = \bigcup_{v_0' \in V_0'} MV_{stop}(v_0') \text{ (Equation S2)}$$
